# Yield of yearly routine physical examination in HIV-1 infected patients is limited: A retrospective cohort study in the Netherlands

**DOI:** 10.1371/journal.pone.0179539

**Published:** 2017-06-21

**Authors:** Marleen A. van Amsterdam, Sander van Assen, Herman G. Sprenger, Kasper R. Wilting, Ymkje Stienstra, Wouter F. W. Bierman

**Affiliations:** 1 Infectious Diseases Service, Department of Internal Medicine, University Medical Center Groningen, University of Groningen, Groningen, the Netherlands; 2 Department of Internal Medicine, Treant Zorggroep, Emmen, the Netherlands; 3 Department of Medical microbiology & Infection control, University Medical Center Groningen, University of Groningen, Groningen, the Netherlands; University of California Los Angeles, UNITED STATES

## Abstract

**Background:**

Routine physical examinations might be of value in HIV-infected patients, but the yield is unknown. We determined the diagnoses that would have been missed without performing annual routine physical examinations in HIV-infected patients with stable disease.

**Methods:**

Data were collected from the medical records of 299 HIV-1-infected patients with CD4 count >350 cells/mm^3^ if not using combination antiretroviral therapy (cART), or CD4 count >100 cells/mm3 and undetectable viral load if using cART. We defined the diagnoses that would have been missed without performing routine physical examinations on annual check-ups in 2010. Exclusion criteria were hepatitis B/C co-infection, start/ switch of cART < 24 weeks, pregnancy, and transgenderism.

**Results:**

215 patients (72%) had positive findings: lipodystrophy (30%), lymphadenopathy (16%) and hypertension (8.4%) were the most common. Two-thirds of all findings were not new or were based on complaints indicating a physical examination even if not routinely scheduled. For 24 patients (8.0%) the routine physical examination led to the finding of a new diagnosis: six—all men who have sex with men (MSM)—had a concurrent sexually transmitted infection, eight had hypertension, and ten others had a large variety of diagnoses. A total atrioventricular block with bradycardia was the most clinically relevant finding.

**Conclusions:**

Annual physical examinations of HIV-infected patients with stable disease brought few new diagnoses that would have been missed without performing a routine examination. Our results suggest that standard assessments could be restricted to six-monthly measuring blood pressure in all patients and annually performing anogenital and digital rectal examination on MSM.

## Introduction

Nowadays, life expectancy of HIV-infected patients in the developed world may approach life expectancy similar to the HIV-negative population if diagnosed early and treated effectively [1]. Problems HIV-infected patients currently face are mostly caused by non-AIDS-defining comorbidities and long-term complications of combination antiretroviral therapy (cART) [2–4]. HIV-infection has turned into a chronic disease.

International guidelines advise regular clinical assessment of the HIV-infected patient, yet the exact content and frequency of advised assessments are not clearly defined and practices vary amongst different hospitals [5–8]. An important area of uncertainty is the role and content of physical examination. Recommendations are mostly based on expert opinions as no clinical studies have been performed on this specific subject.

The objective of our study was to determine the yield of yearly routine physical examinations performed on HIV-infected patients with stable disease. The findings from these routine physical examinations and the subsequently performed diagnostic tests and results are presented. We discuss the role of physical examination in HIV-infected patients with stable disease in the early detection of comorbidity, in particular the detection of malignancy or other serious conditions potentially leading to death.

## Materials and methods

### Study design and participants

Retrospective data were collected from all HIV-1 infected patients who visited the HIV-outpatient clinic from the University Medical Center Groningen (UMCG), a tertiary care center in the Netherlands, in 2010. Patients were included if a follow-up of at least one year was available and if they had stable disease. The findings of the yearly routine physical examination performed by the infectious diseases (ID)-physician were recorded, as well as the diagnostic tests and diagnoses resulting from these findings. All patients’ files were checked for a new diagnosis of a malignancy, cardiovascular event or death in the period of 2010 till November 2014. In order to complete our data on demographics, medical history, malignancy and death we consulted the database of the observational AIDS therapy evaluation in the Netherlands cohort, maintained by the Stichting HIV Monitoring (SHM). SHM is the national executive organization for the registration and monitoring of consenting HIV-infected patients registered for care in one of the 27 Dutch HIV-treatment centers [4].

The ethics review board of the UMCG (METc UMCG) reviewed and approved our study protocol concluding that it was in accordance with Dutch Law because of its retrospective nature (METc number 2011/128).

#### Stable disease

In our outpatient clinic stable disease was defined differently for patients with or without use of cART. For patients not using cART, their disease was considered stable if they had a CD4+ T-cell count (CD4 count) ≥ 350 cells/mm^3^, regardless of viral load. Patients with a CD4 count < 350 cells/mm^3^ were still considered stable at time of visit if their preceding CD4 counts were all ≥ 350 cells/mm^3^. The criterion of 350 cells/mm^3^ was chosen in accordance with the prevailing national guidelines for the initiation of cART at the time of the study period [7]. For patients using cART, disease was considered stable if their CD4 count was ≥ 100 cells/mm^3^ in combination with an undetectable viral load (HIV-1 RNA plasma concentration < 40 copies/ml). Patients with a single detectable viral load followed by an undetectable viral load were considered as having a viral blip and were included [9,10].

Patients with hepatitis B or C co-infection, transgenderism, and patients with extra visits to the outpatient clinic due to new physical complaints, pregnancy, and recent start or switch of cART (< 24 weeks) were excluded.

#### Procedures at the outpatient HIV clinic

The earlier defined stable HIV-infected patients visit one of the two nurse practitioners twice yearly, and one of the five ID-physicians once yearly. Besides a detailed interview and laboratory testing including haematology, blood-chemistry, CD4 count and viral load, these check-ups include a thorough physical examination. This examination includes measurement of the blood pressure (140/90 mmHg regarded as cut off for hypertension), pulse and weight, inspection of the mouth, oropharynx, head, neck, lymph nodes, heart, lungs, abdomen, skin and extremities. The physician performs a digital rectal examination of men who have sex with men (MSM). Inspection of the urogenital tract of women and heterosexual men is not standard. MSM are screened for syphilis at least once a year. Screening for *Chlamydia trachomatis* and *Neisseria gonorrhoeae* (by oropharyngeal, urethral and rectal swab) is not a standard procedure, but is recorded if performed. In the Netherlands, screening for sexually transmitted infections (STIs) in HIV-infected patients is provided by the STI Outpatient Clinic of the national Public Health Service and the general practitioner. Female HIV-infected patients visit a gynaecologist to screen for cervical (pre-) malignancy yearly. Results, conclusions and plans are summarized in correspondence to the general practitioner.

### Data collection and analysis

Subjects were anonymized by numeric encoding. Subjects with positive findings at physical examination were split up in different subgroups, based on the character of the physical finding, the medical history for that particular finding (whether or not it was already known from previous investigations), and the presence of reported and related complaints during the history taking. Patients with new physical findings leading to further investigations and active follow-up were described in more detail. For data analysis, SPSS 22.0 (IBM IBM Corp., Armonk, New York) was used. Only descriptive analyses were performed due to the small numbers of patients with new diagnoses and the heterogeneity of these new diagnoses.

## Results

**Fig 1:**
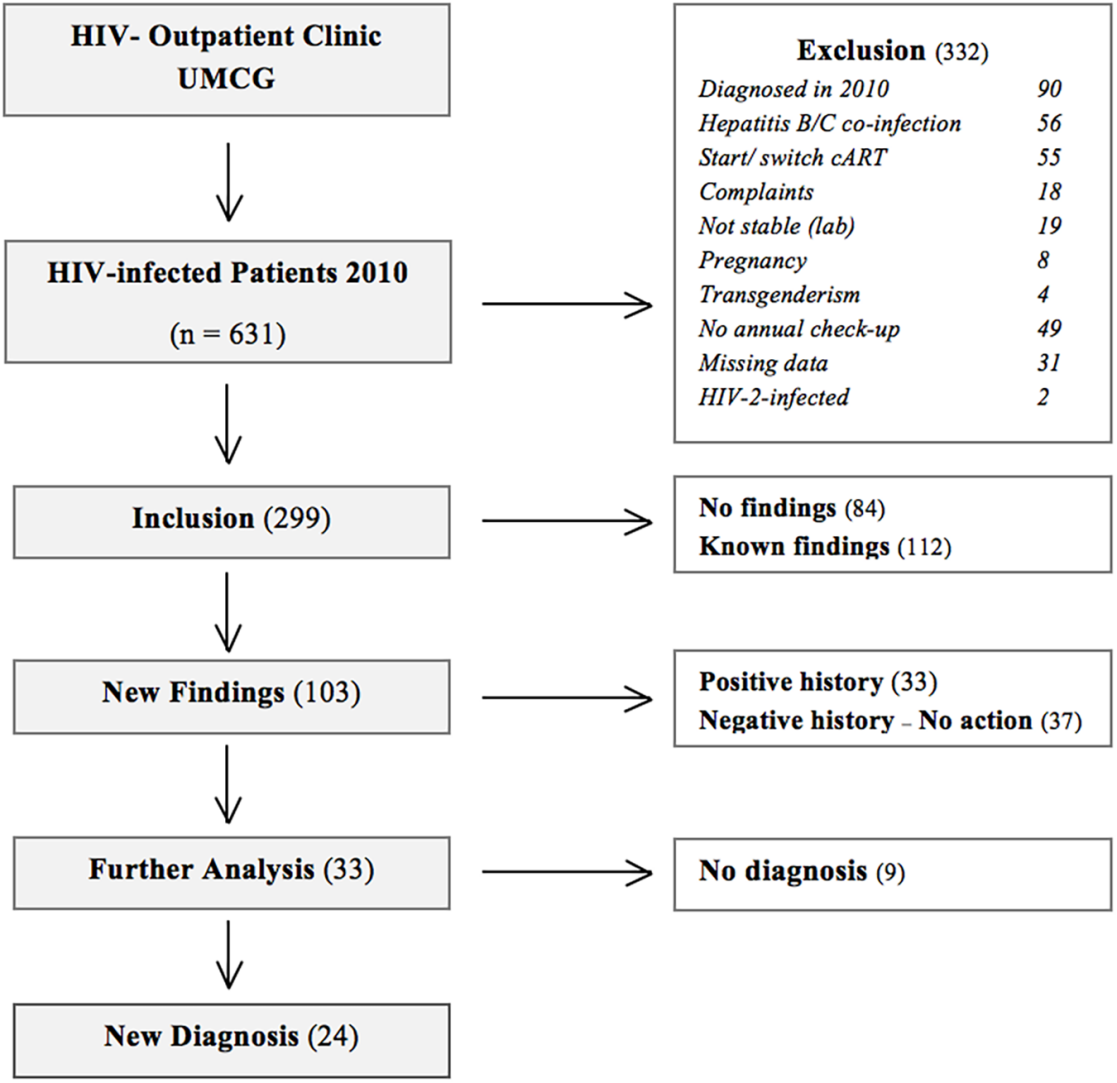
Flowchart patient selection.

Six hundred thirty-one patients visited the HIV-outpatient clinic in the UMCG in 2010. We excluded 252 patients who did not meet our inclusion criteria, and 31 patients for missing data. Forty-nine others did not have an annual check-up in 2010 because of various reasons, mostly because they did not show up or rescheduled their visits. Finally, 299 HIV-1 infected patients were included in our study. Fig 1 shows the results of our selection procedure. Table 1 shows the characteristics of the study population. Most patients were male (81%) and the majority received cART (82%). The median CD4 count was 580 cells/mm^3^.

**Table 1:** Patient characteristics.

	n (%), median (IQR)	
	All Patients (n = 299)	New Diagnosis (n = 24)
Demographics		
Male	243 (81.3%)	22 (91.7%)
Age, yr	47 (39–55)	50 (34–60)
Region of Origin		
The Netherlands	221 (73.9%)	20 (83.3%)
Other Western- Europe	8 (2.7%)	1 (4.2%)
Sub-Saharan Africa	38 (12.7%)	1 (4.2%)
Caribbean	14 (4.7%)	1 (4.2%)
Other/ Unknown	18 (6.0%)	1 (4.2%)
Transmission		
MSM	179 (59.9%)	9 (79.2%)
Heterosexual	96 (32.1%)	13 (12.5%)
IDU	3 (1.0%)	0
Blood products	2 (0.7%)	0
Other/unknown	19 (6.4%)	2 (8.3%)
Years aware of HIV	8 (4–12)	8 (4–12)
HIV infection stage*		
Stage 1	22 (7.4%)	5 (20.8%)
Stage 2	121 (45.5%)	12 (50.0%)
Stage 3	156 (52.2%)	7 (29.2%)
ART history		
Years on ART	8 (4–12)	9 (6–15)
Therapy		
none	55 (18.4%)	8 (33.3%)
1^st^ line	201 (67.2%)	15 (62.5%)
2^nd^ line	30 (10.0%)	0
salvage	13 (4.3%)	1 (4.2%)
cART		
none	55 (18.4%)	8 (33.3%)
NNRTI-based	148 (49.5%)	13 (54.2%)
PI-based	60 (20.1%)	1 (4.2%)
3 NRTI	5 (1.7%)	1 (4.2%)
other	31 (10.4%)	1 (4.2%)
Laboratory		
CD4 count (cells/mm^3^)	580 (440–740)	620 (230–1440)
HIV1-RNA (copies/ml, patients not on cART)	16,400 (3,600–58,000)	28,700 (2,610–122,525)
Smoking		
Current	103 (34.4%)	12 (50.0%)
Ex-smoker	63 (21.1%)	4 (16.7%)
Never	52 (17.4%)	4 (16.7%)
Unknown	81 (27.1%)	4 (16.7%)
BMI, kg/mm2		
<20	41 (13.7%)	2 (8.3%)
20–25	155 (51.8%)	8 (33.3%)
25–30	70 (23.4%)	14 (58.3%)
>30	20 (6.7%)	0
Unknown	13 (4.3%)	0

CD4 count (cells/mm3) and HIV1-RNA (copies/ml) are measured at or shortly before the annual check-up in 2010. Years on ART refers to the initial start of ART. ART = antiretroviral therapy. BMI = body mass index. cART = combination antiretroviral therapy. IDU = injecting drug use. MSM = men who have sex with men. NNRTI = non-nucleoside reverse transcriptase inhibitor. PI = protease inhibitor.

* According to the 2014 revised surveillance case definition for HIV infection (United States) of the Centers for Disease Control and Prevention [11].

### Physical examinations

Two hundred fifteen patients (72%) had positive findings at physical examination. Two-thirds of these findings were already detected by earlier physical examinations (n = 112) or could be related to current complaints (n = 33).

The most common findings amongst all included patients were lipodystrophy (n = 89), lymphadenopathy (n = 48), and high blood pressure (n = 60). In 13 patients’ files the size of the nodule(s) were described as ≥ 1 cm. For one patient the lipodystrophy was a new (self-reported) finding and led to a switch in his cART regimen. Lymphadenopathy was new for 9 of the 48 patients, but not considered suspicious for a malignancy or co-infection by the ID-physician. No biopsies were performed. None of these 48 patients turned out to have a malignancy by the end of 2014. Twenty-six of the 60 patients with a high blood pressure had a medical history with hypertension. For 9 patients the high blood pressure was explicitly recorded as a new finding: 8/9 patients had repetitive abnormal blood pressure measurements and were in the group with new diagnoses. The other 25 patients either had normalized blood pressures by the end of 2011 (n = 14), or the decision to start treatment was still pending due to varying blood measurement results (n = 11).

Thirty-seven patients had new, unmentioned, findings that did not have any further consequence. Amongst these findings were gingivitis, poor dental condition, incisional hernia, enlarged prostate, anal skin tags, eczematous, and simple papular skin lesions.

Thirty-three of the included 299 patients had 36 new and unmentioned findings that led to an additional diagnostic workup. Table 2 shows these particular findings, the subsequently performed diagnostics and results. Nine of the 33 patients had new findings that led to a diagnostic workup without the need for further intervention or treatment. For 24 patients (8.0% of total study population) these diagnostic procedures revealed new diagnoses.

**Table 2:** New findings and negative history: further investigations and diagnoses *(n = 33)*.

*Tract*			
Finding	Investigations	Diagnosis	Treatment
*Head/neck*			
Swelling tongue	Biopsy	Verrucous hyperplasia + fungal infection	Excision
White plaque on tonsil	Culture	Candida	None
Perleche	None	Perleche (empirical)	Acyclovir
*Cardiovascular*			
High blood pressure (n = 8) *	24RR (n = 2), ECG, chest X-ray, referral GP (n = 6)	Hypertension	Antihypertensive drugs (n = 7)
Bradycardia	ECG	Total AV-block	Pacemaker, ceftriaxone
*High blood pressure (n = 3) †*	*ECG (n = 2)*, *referral GP (n = 2)*	*None*	
*Cardiac murmur*	*ECG*, *ultrasound*, *X-ray*	*None*	
*Lungs*			
Reduced breath sounds	Chest X-ray	N. phrenicus paralysis	None
*Prolonged expirium*	*Chest X-ray*	*None*	
*Abdomen*			
Abdominal mass	MRI, ultrasound, Lab: CA-125	Myomatous uterus	Progestativa
*Ascites*	*Ultrasound*	*None*	
*Urogenital*			
Scrotal plaque	Punch biopsy	Cutaneous chromomycosis	Excision + terbinafine
Genital ulcer	Syphilis serology, HSV- 1/2	Syphilis I (reinfection)	Benzathine benzylpenicillin
Enlarged prostate *	PSA, urine culture	Prostatitis	None
Scrotal cyst	None	Atheromous cyst	Surgery
Genital warts #	None	Condylomata acuminata	Cryotherapy
*Rectal*			
Palpable mass	Proctoscopy	Condylomata acuminata	Electrocoagulation
Anal warts	None	Condylomata acuminata	Cryotherapy
Anal ulcer	STI swab, HSV- 1/2	Gonorrhoea + Chlamydia	Antibiotics
Erosive anal defects	STI swab	Gonorrhea	Antibiotics
*Erosive anal defects*	*STI swab*	*None*	
*Anal irregularity*	*Sigmoidoscopy*	*None*	
*Skin*			
Subcutaneous noduli #	Referral dermatology	Naevus naevocellularis	None
Actinic keratosis	Referral dermatology	Actinic keratosis	Cryotherapy
*Dark skin*	*Ferritin (high)*, *genetic testing hereditary hemochromatosis*	*None*	
*Extremities*			
Dermatomycosis (feet)	None	Mycosis	Miconazol
*Exostoses †*	*X-ray*	*None*	

* One patient had both prostatitis and hypertension (treated).

*†* Another patient had both exostoses on his feet and a high blood pressure.

# One patient had both naevocellular naevi and condylomata acuminata.

24RR = 24 hour blood pressure measurement. AV = atrioventricular. CA-125 = cancer antigen 125. ECG = electrocardiogram. GP = general practitioner. HSV- 1/2 = Herpes Simplex Virus 1/2 (DNA). MRI = magnetic resonance imaging. PSA = prostate- specific antigen. STI = sexually transmitted infection. US = ultrasound.

Italic: patients that turned out to have no particularities

As mentioned earlier, 8 patients had hypertension. Six others had an STI: condylomata acuminata (n = 3), syphilis reinfection (n = 1), gonorrhoea (n = 1), or both gonorrhoea and chlamydia (n = 1). Ten patients had various other diagnoses, mostly affecting the oral cavity or the skin. Amongst the patients with new diagnoses was a 63-year old man with a bradycardia of 40 beats per minute. He was immediately referred to the emergency aid and turned out to have a total atrioventricular block of unknown origin. *Borrelia burgdorferi* infection was suspected and the patient was treated with intravenous ceftriaxone for 14 days. Yet, this diagnosis could not be confirmed by immunoblot analysis. The bradycardia persisted and three weeks later a pacemaker was implanted. Another patient with a phrenic nerve palsy was referred to the neurologist, who ruled out neuroborreliosis and neuralgic amyotrophy and considered the palsy to be idiopathic. However, his ID-physician suspected a herpes zoster reactivation to be the cause.

### STI screening

One hundred ninety of the 299 included HIV-patients were screened for STIs by laboratory methods: i.e. for syphilis, chlamydia and/ or gonorrhoea. Thirteen of the 190 patients (11 MSM) had mentioned to be at risk for having an STI. Seven patients had signs of an STI at their physical exam, without having reported symptoms. Motivation for the STI screening of the 172 others was not explicitly reducible from the medical files. In total: five patients (2 MSM) were tested for chlamydia and gonorrhoea, 26 patients (23 MSM) for chlamydia, gonorrhoea and syphilis, and 159 patients (120 MSM) were tested for syphilis only. Test results of all non-MSM were negative. Seven of the 145 tested MSM had an early syphilis re-infection, 6 others had chlamydia, 2 patients had both chlamydia and gonorrhoea, and 1 MSM had gonorrhoea. The latter 3 were amongst the patients who had mentioned to be at risk for having an STI. Thirteen of the 145 screened MSM (9.0%) had STIs without having reported symptoms or risk behaviour, of whom 3 were in the group with new diagnoses.

### Follow-up

Between 2010 and 2014, 4 patients died, 18 patients were diagnosed with a (pre) malignancy, and 4 patients had major cardiovascular comorbidity. Causes of death (out of hospital cardiac arrest (n = 1), diffuse large B-cell lymphoma (n = 1), and unknown (n = 2)) could not be related to any results of physical examination during routine visits. Ten patients had non-melanoma skin cancers, amongst which 8 were basal cell carcinomas. Others had anal dysplasia (n = 2), recurrent anal carcinoma, sigmoid carcinoma, metastatic melanoma, and a relapse diffuse large B-cell lymphoma. Two of the malignancies, i.e. the tonsil carcinoma and carcinoma of the soft palate, were detected at the physical examination. Both patients were symptomatic and mentioned complaints directing towards the diagnosis. The cardiovascular comorbidities were either nonSTEMI (n = 3) or hemorrhagic stroke (n = 1). The latter 4 patients were all asymptomatic at their annual check-ups.

## Discussion

To our knowledge, this is the first study evaluating the yield of routine physical examinations as part of follow-up of HIV-infected patients with stable, chronic disease. We showed that an annual routine physical examination in HIV-infected patients with stable disease revealed few new diagnoses that would not have been found without these examinations. The total AV-block presumably was the only diagnosis of vital importance in the short run. Both other frequent diagnoses, hypertension and STIs, have important clinical relevance in the longer run or are important from a public health perspective.

Frequently diagnosing hypertension corresponds with a higher prevalence of hypertension amongst HIV-infected patients compared to the general population, which is related to traditional risk factors, antiretroviral drugs and lipodystrophy [12–14]. The high incidence of STIs we observed was to be expected and is related to high-risk sexual behavior amongst MSM [15,16]. For STIs in HIV-infected patients self-report of symptoms as a screening method was shown to be unreliable in earlier studies. Heiligenberg et al. found STIs in 16.0% of 659 HIV-infected MSM in the Netherlands who did not spontaneously report symptoms, mostly anal chlamydia and syphilis [15]. Furthermore, Tuddenham et al. aimed to determine the contribution of physical examination in detecting STIs that may not be identified by current laboratory methods, such as early syphilis, balanitis, and genital warts. They found that 2.7–10.4% of the patients would have had missed diagnoses if no physical examination was performed [17]. Importantly, an HIV-infected person with a concurrent STI is more likely to transmit HIV through sexual contact than other HIV-infected persons, especially in the case of genital or anal ulcers or discharge. Adequate treatment of STIs is of public health importance [18]. For example, untreated syphilis can have a great clinical impact because of the risk of late complications such as neurosyphilis. Moreover, syphilis is associated with virological and immunological failure of HIV therapy [19,20]. Finding new condylomata acuminata is of potential importance. Although not causatively related to anal malignancy, condylomata acuminata indicate high-risk behaviour and exposure to *human papillomavirus*. Consequently, they are indirectly associated with anal cancer amongst HIV-infected MSM [21,22]. Digital rectal exam might be helpful in detecting anal carcinoma in an early stage in asymptomatic HIV-positive MSM, therewith having implication for the treatment strategy [23].

Strikingly, no malignancies were detected by performing physical examination per se, despite an increased risk of malignant lymphoma and non-HIV-related cancers in HIV-infected patients on cART [24–26]. The malignancies that were diagnosed in our follow-up period were mostly revealed by specific complaints. New malignancies of the skin were discovered either by the dermatologist during routine follow-up of previous skin cancers, or by the general practitioner. In the general population there is a lack of direct evidence linking skin cancer screening to improved health outcomes [27,28]. Although not studied, this might be similar for the HIV-positive population with stable disease and a (near) normal immune function. High lymphadenopathy rates, without proven malignancy, might be partially explained by the fact that enlarged lymph nodes may persist for many years after primary HIV-infection [29,30].

Our study has some limitations. First, our study population was relatively small and the group of new diagnoses was quite heterogeneous. With a larger study population and longer follow-up, it might have been possible to perform stratified analyses (e.g. by age and gender) to identify categories of patients who do benefit from the routinely performed physical examination. Second, the retrospective design complicates the correct classification of findings as solely detected by the routine examination. This may have led to both an underestimation and an overestimation of the effect of the routine physical examination. Third, the letters to the general practitioners used as one of our data sources were potentially susceptible to interpretation by the investigator. Fourth, determining whether the yield of routine physical examinations is clinically relevant or not is inevitably a subjective judgment. We tried to overcome this problem by seeking consensus among the physicians. Also, in the follow-up period we only looked for diagnoses of less arguable relevance, i.e. cancer, cardiovascular events and deaths. Finally, a minority (18%) of our study population had unsuppressed viraemia, since they were not on cART yet. This was in accordance with the prevailing guidelines for the initiation of cART at time of our study [7], while 2016 guidelines advise cART in all adults with chronic HIV infection [6,31]. However, we still consider our study population a representative sample of the current HIV-infected population, because also in the present not all HIV-infected patients are on cART (for various reasons). Potential adverse effects of the routine physical examination such as false reassurance and costs were not assessed.

In the HIV-negative population, two reviews evaluated the evidence on benefits and harms of general health checks and periodic health evaluation. A potential benefit is a reduction of patient worry [32]. However, even though more diagnoses had been identified in some studies, no clear effect could be found on morbidity or mortality [33].

Although our findings have to be interpreted with some caution, our findings do suggest that a routine thorough physical examination is unnecessary. Performance of a targeted routine physical examination increases time-effectiveness and prevents unnecessary additional diagnostic procedures. On the other hand, for centers where no annual routine physical examination is being performed this would imply a limited, but conceivably valuable extension.

Producing more robust conclusions on the yield of routine physical examination is bounded by the perceived difficulties in defining the threshold of effectiveness of such examination and the different strategies of routine physical examinations between hospitals in the Netherlands. We propose a follow-up study to overcome some of the abovementioned limitations and strengthen our findings. Such a study would have to be multicenter, including centers with different strategies of routine physical examinations, and be limited to outcome measures of which the relevance is hard to debate, e.g. cancer, hypertension and STIs. Based on our results. this would have to be a large study encompassing at least 5 years. A retrospective design could be valid but complicated by several forms of bias.

### Conclusion

For HIV-infected patients with stable disease we suggest to adapt routine examinations to known risks in this population and only perform additional targeted physical examination guided by complaints. In accordance with most guidelines we advise regular, 6-monthly, assessment of weight, blood pressure and BMI. Although few guidelines discuss or recommend it [34], we do advise to perform anogenital inspection and digital rectal exam on all MSM annually, since (asymptomatic) STIs are frequently discovered [15,17], and the treatment provides individual as well as public health benefit [18].

## Supporting information

S1 Data SPSS file containing anonymized data used for presented analyses. To increase anonymity the variable Year of Birth was removed.
(SAV)
